# ‘The Head Carver’: Art Extraordinary and the small spaces of asylum

**DOI:** 10.1177/0957154X16676693

**Published:** 2016-11-10

**Authors:** Cheryl McGeachan

**Affiliations:** University of Glasgow, UK

**Keywords:** Art therapy, asylum, biography, landscape, mental health, outsider art, Scotland, space, surveillance

## Abstract

This paper uses the unique collection of Scottish outsider art, labelled Art Extraordinary, as a window into the often neglected small spaces of asylum care in the early twentieth century. By drawing upon materials from the Art Extraordinary collection and its associated archives, this paper demonstrates the importance of incorporating small and everyday spaces of care – such as gardens, paths, studios and boats – into the broader historical narratives of psychiatric care in Scotland. Examples of experiential memorialization and counterpoints to asylum surveillance culture will be illuminated. The significance of using ‘outsider’ art collections as a valuable source in tracing geographical histories will be highlighted.

## Introduction

The vibrancy of recent scholarship on lunatic asylums and their social ramifications within the history of psychiatry is notable, revealing a fascination with the histories of mental ill health and its component ‘spaces’. For example, various studies have unpicked the foundations of British and Irish institutions, from the chartered royal hospitals (Andrews et al., 1997; Prior, 2012; Walsh, 2012) to the more unknown Scottish district asylums (Ross, 2014), demonstrating their importance in the conceptualization of ‘madness’ and mental health care in different times and places. Scull’s (1979, 2006, 2015) calls to expose the range of economic, social, political and cultural dimensions to ‘madness’ has inspired research into the spatial distribution of asylums (Dear and Wolch, 1987; Philo, 2004), networks of the psychiatric profession (Andrews and Smith, 1993; Miller, 2004), practicalities of ‘asylumdom’ (Davis, 2008; MacKenzie, 1992) and individual practitioner and patient case studies (Beveridge, 2011; McGeachan, 2014). Significant work has also opened up the smaller sites and spaces of the asylum, including laboratories (Finn, 2012), cemeteries (Philo, 2012), sports facilities (Ellis, 2013), and specialist sites for treatment such as insulin coma wards (McGeachan, 2013) and scientific intervention such as post-mortems (Andrews, 2012; Wallis, 2013). Attention to the differing practices of surveillance in these ‘small spaces’ has also arisen, highlighting the configurations of power bound up with such institutional nooks and crannies (Hide, 2014; Monk, 2008). This work has deepened the understanding of mental health care in different periods, allowing the experiences of individuals caught up in these ‘care’ systems to seep out of the asylum and into wider histories discussing mental illness, health, creativity, medicalization and well-being.

The multiple lives of those living and working in and creating asylum spaces have been documented, but in many of these studies the patient’s own experience still remains eerily silent or portrayed only through the lens of clinical discourse. Despite attempts to unearth the patient experience (Porter, 1987), certain voices, and therefore the experiential stories of being in certain places, remain difficult to hear and continue to challenge the historian’s practice. Recent work on the nineteenth-century asylum system has sought to progress such debates by signalling the importance of exploring boundaries between history and narrative, fiction and fact in the telling of asylum stories (Knowles and Trowbridge, 2014). While previous studies have focused almost exclusively on the written words of psychiatric patients from letters, novels or poems, a small but growing body of work is turning towards the ‘art of the insane’ to reveal the patient experience differently (Beveridge and Williams, 2002; MacGregor, 1989).

In this vein, the present paper seeks to examine a Scottish collection of the art of the insane, labelled ‘Art Extraordinary’. Focusing on artwork created by one individual in Scottish psychiatric care from 1901–50, attention is drawn to the personal and collective inhabitation of small spaces often neglected in the wider narratives of institutional life. Using a set of patient artworks as the focus allows a different set of patient experiences to come to the fore, experiences currently lost or unheard in the stories of the insane to date.

## (In)sanity, art and Scotland

Scotland holds a distinctive claim to the art of the insane in the collection of patient work amassed by Dr W.A.F. Browne, the first Physician Superintendent of Crichton Royal Institution in Dumfries 1838–57 (for accounts of Browne’s influence, see Park, 2010; Scull, Mackenzie and Hervey, 1996; Williams, 1996). Under Browne’s ‘moral health’ regime, Crichton patients were stimulated by artistic and other cultural activities designed to alleviate, or potentially to cure, their mental distress. Browne’s collection of ‘pseudo art’ is possibly the first of its kind in the world, with ‘no other known earlier example of asylum art being fostered, collected and valued in such a comprehensive manner’ (Park, 2010: xvi). Unlike the sporadic cases reported at Bethlem, the making of art was built into Crichton’s daily regime, with patients encouraged to draw as a means of ‘displac[ing] that brooding melancholy which the monotony and idleness of confinement so often engender[s] in the active and educated mind’ (CRI [papers], in Park, 2010: 33). Browne’s influence on the Scottish asylum system was substantial, manifested by his role as Commissioner in Lunacy 1857–70 following the 1857 Scottish Lunacy Act (Andrews, 1998) and his training of many future Scottish asylum superintendents.

While Browne was demonstrating that ‘sane’ work could be created by the ‘insane’, various individuals working in mental hospitals across Europe became interested in collecting and displaying the art of the insane for their research on the inner worlds of their patients. Examples include the Swiss psychiatrist Walter Morgenthaler, whose book *A Mental Patient as Artist* (1921/1992) details the work of Adolf Wölfli, arguably the best-known outsider artist of all time (Park, Simpson-Housley and De Man, 1994). Perhaps the most influential psychiatric art critic, though, was Hans Prinzhorn, a German working at the Heidelberg Hospital, whose book *Artistry of The Mentally Ill* (1922/1972) explored the artwork of disturbed psychiatric patients for links between creativity, irrationality and sickness. He argued that, rather than simply inspecting these pieces for signs of insanity, they could be approached as works holding aesthetic value. All these studies, and numerous others, took seriously the artistic work of psychotic patients, addressing the different aspects of the clinical and psychological experiences revealed. Yet, as Parr (2008: 137) contends, ‘through this process patients’ own representations were … subsumed into a clinical framing of disorder, one that resonated with the difference-making of other medical visualisations’.

Psychiatrists have primarily valued this work within its clinical confines for what it reveals about the condition of mental illness, but historians have begun to consider what patient art can reveal about the creator and his/her experience, particularly pertaining to the asylum world from which it often came (Beveridge and Williams, 2002). This paper uses the Art Extraordinary collection of patient art to examine the different institutional sites where this type of art was being produced, the purpose being to shed light on the complex histories and geographies of mental health care in Scotland bound up with the art of the insane. Beginning by discussing the making and complexities of the Art Extraordinary collection, the deep-rooted links between art, mental health and collecting to Scottish asylum histories are flagged. Then, by closer investigation of the art of one patient, Adam Christie – affectionately termed ‘The Head Carver’ (Keddie, 1984: 14) – a range of under-examined small spaces of asylum care, such as gardens, paths and studios, creep into view. Throughout, issues of asylum surveillance and its subversions are addressed, offering important counterpoints to the surveillance culture of asylumdom. Finally, by concentrating on the art itself as revealing the stories of the insane, the significance of using outsider art collections for casting fresh light on hidden asylum histories is clarified.

## Art Extraordinary

Terminology surrounding the art of the insane can be incredibly problematic.^1^ When discussing outsider art, Cardinal (1972: 179)^2^ suggests: ‘An alternative art exists. It need not be geographically remote, nor need it have a single location. It crops up in all places where Art is considered to have no place.’ The domain of such art is therefore a tangible one, but specifically demarcates the artists as somehow different from their audience, beyond the parameters for normality set by the dominant culture (Rhodes, 2000: 7). Similarly to outsider art, the term Art Extraordinary is deeply indebted to the French artist Jean Dubuffet, who coined the term ‘Art Brut’ in the 1940s, depicting a raw, spontaneous type of direct art and inviting the world to look for ‘true art’ in unexpected places (Rhodes, 2000: 23). Cardinal (1972: 179) stresses: ‘If art brut is not a static place on the map, it can be a direction. Its rare light beckons towards an impossible horizon where the most intensely human merges into the abhuman.’

Dubuffet first searched for true art in drawings by children, but, inspired by Prinzhorn, he turned to psychiatric patients and began collecting art by asylum inmates (Rhodes, 2000: 43). Dubuffet’s hunt for raw art reflected a quest to identify what lies in contrast to culture, an oppositional position with which he felt the mentally ill were attuned, and he supposed: ‘It may be that artistic creation, with all that it calls for in the way of free inventiveness, takes place at a higher pitch of tension in the nameless crowd of ordinary people than in the circles that think they have the monopoly of it’ (Dubuffet, quoted in Laing, 1996: 11). In characterizing Art Brut, Dubuffet struggled and declared that ‘no common definition will fit these works … [each] being invented by the maker for his own purposes of the moment’ (in Laing, 1996: 11).

Delineating her own boundaries, the pioneering Scottish art therapist and collector of patient-art Joyce Laing defined Art Extraordinary as visual art forms created by individuals who paint, sculpt, weave, sketch or draw due to a *compulsion* to express an intense personal vision. Most of these artists have no formal art education and often exist on the margins of conventional society, held within the care of institutions and frequently experiencing mental health difficulties of varying kinds. The compulsive nature of this creativity means that there is no desire to create for commercial gain, many pieces being discarded by the individuals who make them as soon as they are finished. Due to these constraints, Art Extraordinary is deemed very rare and can be difficult to source and to trace.

When discussing the art of the insane it is essential to confront the figure of the collector, the individual who chooses to gather together and store selected objects for a wide set of personal, cultural and social reasons. Recent studies of collecting consider the physical connections produced and cultural values represented in the historical linkages between people and things (Dudley, 2012). The entanglement of objects and collectors within a broad set of social relations thus comes into view, emphasizing the importance of retelling the narratives of both objects and the persons with whom they were once associated (Dudley, 2012: 1). The process of selection is central to the collecting and is intimately bound to conflicting notions of value. For Pearce (1995: 27), ‘Collections occupy a particular position in the processes by which value is created, because value is, to a considerable extent, a creation of the imagination rather than of need; and in the play of the imagination the objects themselves are powerful actors’.

A set of powerful critiques have been directed at the collectors of art of the insane over how values are placed on the individual producing the work, as well as on the work itself. Scholars such as Allderidge (2000), Rhodes (2000) and Parr (2008) have questioned how patients’ own representations are frequently incorporated into a clinical framing where the art is portrayed primarily as the product of a specific clinical disorder, rather than being seen as subject to a host of other possible contextual influences. Instead, such scholars emphasize the multi-layered meanings that can be given to such art objects by taking seriously the contexts central to their making, storage and indeed collection. These authors also address the possibilities opened up when the ‘object biographies’ of these artworks (Dudley, 2012: 1), with the (often conflicting) stories that such objects come to embody, are brought into sharper focus.

The Art Extraordinary collection is intimately bound to the life and work of Joyce Laing. Laing, an alumnus of Aberdeen Art College, became the first psychiatric art therapist in Scotland. Beginning in the tuberculosis wards of Glen O’Dee sanatorium at Banchory in the late 1950s, then moving to the role of art therapist in the 1960s at the Ross Clinic in Aberdeen, Laing became part of a dynamic team including psychologists, psychiatrists, nurses and social workers striving for insights into the experiences of patients with mental illness. Laing wished to show the medical team ‘the visual side of madness’ (Laing, quoted in Hutchinson, 2011: 115). Recalling an encounter with one highly disturbed patient at the Ross Clinic, Laing (quoted in Hutchinson, 2011: 116) argues that the paintings of this individual were a vehicle for seeing into his condition:

So the pictures were hallucinations … Medicine had never seen this before. With the psychiatrists talking and talking to them they were getting a verbal description. But they weren’t seeing. And this guy, who was skilled in painting, was actually producing it visually.

For Laing, this way of *seeing* mental ill health was influential, compelling her to explore the connections between creativity and psychiatry in a range of different guises throughout her career.

Inspired by the embroideries and paintings of one patient in an Aberdeen psychiatric hospital, Antonia Jabloner, and by reading Dubuffet, Musgrave and Cardinal, Laing vowed to seek out more of this ‘true art’ in Scotland. When discussing the collecting of Art Extraordinary, she recalls:

I have searched for an art which has the ability to amaze me, accustomed as I have become to seeing strange and wonderful paintings by many patients. Works I classify as Art Extraordinary have … the power to enchant and seduce me into this other sphere for which there is no comparison. (Laing, 1996: 12)

However, as highlighted previously, ‘the lines between people and the objects with which they surround themselves are not always clear’ (Walklate, 2012: 13); also, although beyond the scope of this paper, it is important to note the broader context of Laing’s collecting. While not always explicitly stated in her work to date, it is clear that Laing’s desire to recover endangered forms of Scottish outsider art speaks to a wider agenda, inspired by Dubuffet and others, to challenge exclusionary stigmas within what constituted art in other genres and contexts. For Laing, outsider art has the capacity to disclose another dimension of insight into ‘art forgotten’, and the artists concerned ‘have an ability to span the centuries and … stand like prophets forcing us to re-examine our aesthetic values’ (Laing, 1998: 18). It is also about the people making the art themselves, however, and Laing’s unwavering professional and personal commitment to individuals experiencing mental ill health always drew her to spaces of psychiatric and psychological care. Alongside photographer Jim Waugh, she undertook an expedition in the 1970s to search the wards of Scottish psychiatric facilities for Art Extraordinary, it being decided that ‘we’d start at the north of Scotland and work our way south. So Craig Dunain was our first call’ (Laing, quoted in Hutchinson, 2011: 118). This collecting continued for over 30 years, during which Laing searched in hospital wards, sheds, gardens and rubbish bins to find pieces which fitted her definitional framework of Art Extraordinary. For Blom (2003: 45), collecting comprises ‘an attempt to make sense of the multiplicity and chaos of the world, and perhaps even to find in it a hidden meaning’; and for Laing a part of the collecting process was attempting to understand more about the asylum spaces where art could be made, discarded, hidden and recovered.

Throughout this collection process, Laing located objects made out of woven grass by Angus McPhee (Hutchinson, 2011; Parr, Philo and Burns, 2003), stone sculptures by Adam Christie (see later), animal paintings by an unknown Victorian patient, fantasy landscape paintings by Mrs McGilp, ceramics by Kenneth Annat, photo-collages and coffee jars by Marylene Walker, embroideries by Antonia Jabloner, paintings representing popular culture by Robert A, and landscape paintings by Lachie Kilmichael. Although a few other outsider artists were discovered during the expedition, it is only these individuals that so far earn Laing’s descriptor ‘artists extraordinaire’ due to their compatibility with Laing’s aforementioned definition. The objects and writings forming Laing’s collection span significant and varying dates (*c.*1850–2012), asylums, hospitals and different community spaces of care, illness and recovery. This paper uses pieces from the Art Extraordinary collection to reveal stories that highlight patients’ experiences of these shifting Scottish asylum worlds. By turning to one patient’s stone sculptures and following the stories of the stones, I portray different elements of the patient’s/artist’s experience. By using the stone head sculptures themselves as a window into the Montrose asylum, and also into Adam’s experience of being in this place, hidden stories and under-examined small spaces come into view. Various sources are used in tandem with the artwork – case note records, asylum reports, oral histories, poems and photographs – to capture something of Adam’s asylum worlds.^3^

## Adam Christie and the stories of stone

Huddled, almost out of sight, in the corner of the hospital gardens the distinctive figure of pauper patient, Adam Christie, was turning discarded stone into recognisable shape through a set of unorthodox tools. A heavy file for a hammer and a six inch nail for a chisel moulded inanimate stone into almost-humanlike form. Finishing touches were delicately added by slowly scraping the skin of the stone with a fragment of broken glass chipped from a discarded bottle, bringing his heads almost to life.^4^

**Figure 1.**
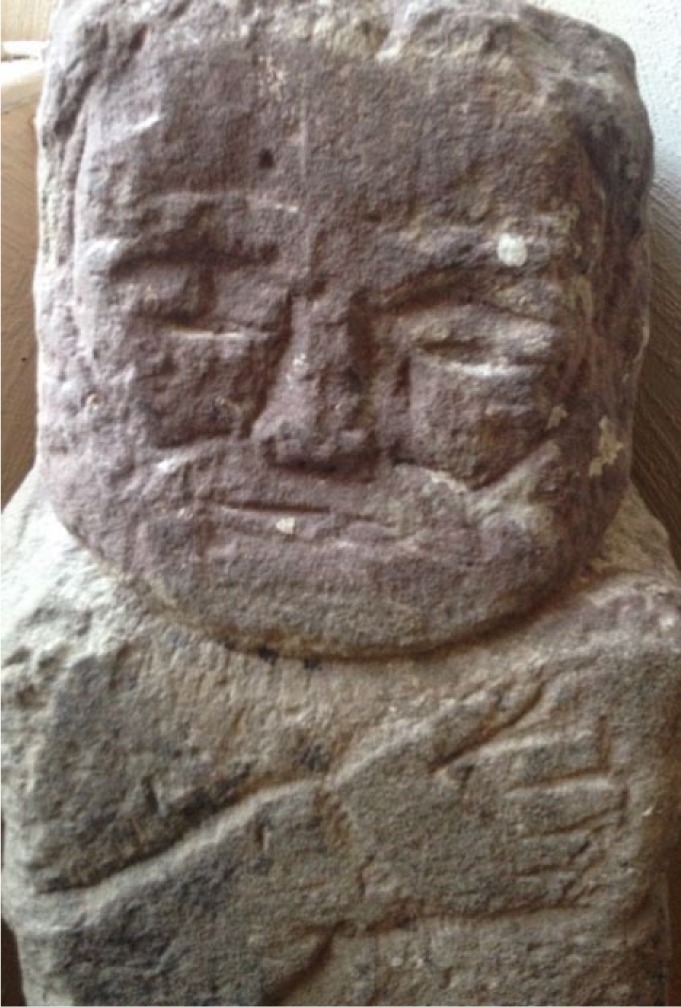
One of Christie’s head sculptures (courtesy of Graeme Lamb, 2015).

The stone heads (see e.g. Figure 1) carved by Adam during his 50 years of incarceration at the Royal Asylum of Montrose are important starting points for recreating Adam’s asylum experience, hinting beyond the asylum walls and into a range of spaces elsewhere that were significant to him. They suggest complex connections between time and space, as Paton (2013: 1077) highlights in relation to the laboured action of a stone–metal–flesh dialogue which fuses relations between past and present. Each of the stone heads represents flows between (geological) time and human relations with place, an intricate relationship between the body and the material (Paton, 2013: 1072).

Adam was admitted to the Royal Asylum of Montrose, frequently referred to as Sunnyside Hospital (Presly, 1981), in 1901 at his family’s request. Previously Adam had become increasingly withdrawn, developing a state of agitation which his family felt left them no alternative but to seek asylum for him (Keddie, 1984: 48). Once admitted, he was diagnosed with ‘delusional insanity’, with records stating that he consistently contemplated committing suicide, felt persecuted and frequently refused food (THB23/4/1/22). Adam, meanwhile, described his own state of mind thus:

I have an excess of nervous energy, sometimes positive, sometimes negative, which reverses the action of my heart … my nervous energy is at present polarised but if it comes into contact with a nature other than my own, it will be perfect pandemonium. (TBH23/4/2/1)

**Figure 2.**
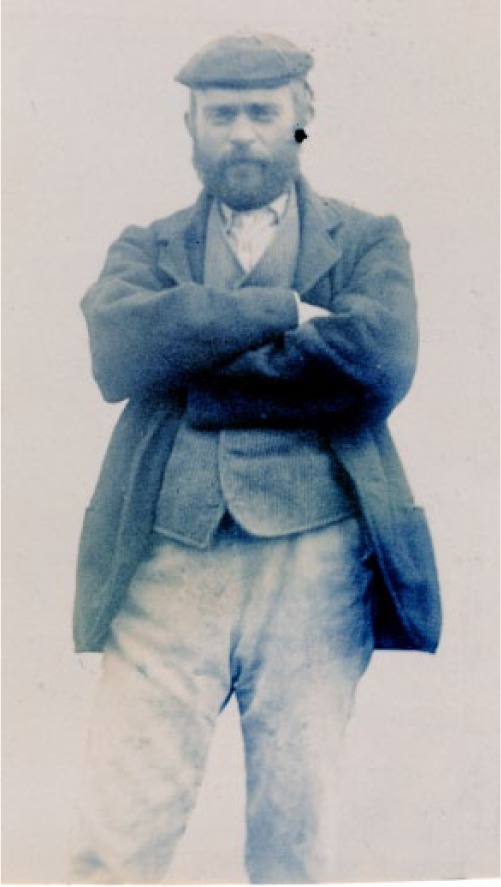
Image of Adam Christie, aged 32, at the Montrose Asylum in 1901 (University of Dundee Archives, THB23/5/3/16: 29).

Throughout his incarceration, Adam had cycles of recurring illness, including ideas of persecution by the Kaiser during World War I; but for much of the time he appeared relatively well and was able to undertake work in the hospital, such as tending gardens and delivering post. On admission, Adam’s photograph was taken by the Clerk of Works Mr W.C. Orkney (Figure 2), capturing something of the Shetlander character that was to become a ‘weel kent’ figure within the hospital community (Keddie, 1984: 63). Adam died in Sunnyside on 7 May 1950, being buried in a pauper’s grave, and through ‘time’s slow erosion’ (Brown, 1984: 8) was largely forgotten as mental health provision in Scotland took increasingly new forms.

Laing (1996: 16) remarks that ‘behind so much of this kind of art are the stories of chance discoveries, which makes one realize how much must have been lost over the years’. Similarly, Rhodes (2000: 22) argues that ‘the discovery of Outsider Art relies on the chance encounter’ with something ‘possess[ing] a tantalizing and fragile presence’. During Laing’s search, this ‘fragile presence’ was often the case, and many of the pieces owed their continued existence to their very lack of noticeability. Their survival depended precisely on being overlooked in their immediate asylum worlds, maybe wholly hidden or perhaps seen but not viewed as either rubbish to be cleared or somehow problematic, effectively ‘hiding in plain sight’ from those who frequented these spaces on an everyday basis. Laing (1996: 15) recalls how a ‘chance remark, a hundred miles from Montrose’, led her to unearth Adam’s stone heads for her collection, causing her search to merge with that of Kenneth Keddie, consultant psychiatrist at Montrose, in a quest to rescue Adam’s story from historical erosion.

Keddie relates that he first came across the stone heads shortly after taking up his post as psychiatrist in 1966. The heads, placed in gardens and peeking out of the hospital shrubbery, had become part of the everyday environment, barely noticeable to those used to the hospital grounds. At this point, few staff remembered who had carved the stones, and it became clear to Keddie (1984: 15) that Adam’s ‘name had been erased from people’s memories’. Keddie and Laing met in the wards of Sunnyside in the 1970s, and Laing urged Keddie to glean everything he could about the mysterious stone-carver before the evidence disappeared forever. Inspired by Laing’s passion to preserve the fragments of lives lived in such places, Keddie conducted substantial research and in 1984 published *The Gentle Shetlander*, detailing Adam’s life and his time as an asylum patient.

## Gardens and dens: no stone unturned

**Figure 3.**
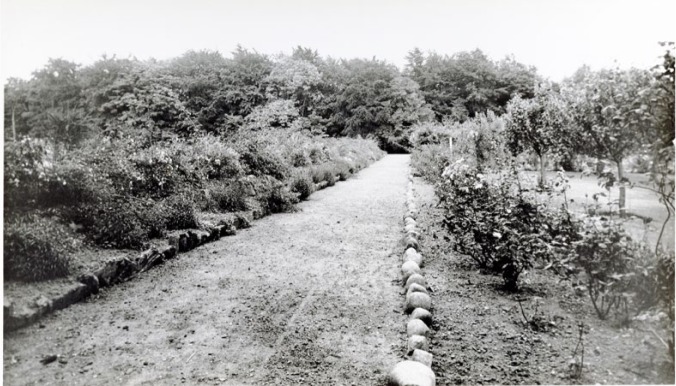
One pathway in the hospital gardens in 1939 (University of Dundee Archives, THB23/19/2).

Beneath the thick, dark foliage creaking in the north-easterly Scottish winds lie the materials for the making of Adam’s art. Neatly piled stones demarcate pathways from garden spaces throughout the hospital grounds, and scatterings of unused and unwanted rubble litter the green space encircling the main hospital building (Figure 3). The first lunatic hospital in Scotland, Montrose Lunatic Asylum, was founded in 1781 and was built on the Montrose Links (Poole, 1841). In line with the core asylum-locational arguments of the nineteenth century (Ross, 2014), an improved asylum was opened in 1878 on a more rural site away from centres of population. Sited on the outskirts of Montrose, in the village of Hillside, the farmland of Sunnyside became an important part of the asylum landscape and shaped, as much as it was shaped by, the people who encountered these sprawling external spaces (Presly, 1981).

The hospital records frequently reported that Adam was busy with his hands. Whether playing the organ, making fiddles, writing letters home or carving in wood and stone, he was clearly compulsive in his artistic creation. At the age of 55, 22 years after entering the asylum, Adam developed an obsessive interest in carving stone, with the hospital grounds being where he could source his materials. Laing (1996: 12) observes that: ‘Artists Extraordinary are all self-taught and use whatever materials they can find provided these suit their needs. They may appear to use inferior scrap but they are excessively particular about each fragment they accept.’

The expansive gardens and farmland became important external spaces where Adam could find materials and create his head carvings, while his unofficial role as hospital postie allowed him to become familiar with pathways through the grounds and the surrounding area. The winding route-ways around the grounds offered fertile pickings for the collection of discarded stone or scraps of materials, such as glass, to use as sculpting tools. Adam would regularly collect the mail from the local Post Office in the village and deliver it, morning and afternoon, to homes around the hospital (Keddie, 1984: 62). Carrying the post in a large bag slung across his shoulder with a strap made of string, this receptacle became the means through which he could carry his tools and be free to sculpt whenever stone became available. Frequently, between deliveries he would perch on a wall or fence to carve his heads from the foraged stone. Many of the walls became part of the creative process, with Adam carefully removing slabs of stone from the wall only to replace them later with a stone carving.

The grounds of the hospital furnished a wide variety of convenient hiding places for Adam’s stonework. Having completed a selection of his peculiar stone creations, he would pile them into a wheelbarrow and distribute them around the hospital grounds, weaving his way through the foliage corridors connecting the external spaces of the asylum. Hiding the heads in shrubbery or between cracks in walls was commonplace, allowing them to become almost invisible features of and in the asylum landscape. The grounds also afforded solitude and privacy to Adam, since he hated to be overlooked while working, and the internal spaces of the asylum were known for their lack of individual space. Embarrassed about surveillance when anyone walked past, he would stop what he was doing and look down at the ground, only resuming when the potential observer was gone (Keddie, 1984). Goffman (1961) introduces the notion of ‘free’ spaces in the asylum, ‘the micro-geographies of reduced surveillance’ (Parr et al., 2003: 349), highlighting where respite from the medical/psychiatric gaze may be found. These free spaces of possibility are often likened to dark corners where illicit and immoral practices are played out, such as drug-taking or sexual activity (Philo, 2007), but they can also be spaces where the asylum landscape becomes a meeting place between the inner and outer self: a liminal space ‘in-between’ (Bondi, 2005). This tension between being seen and feeling hidden highlights the curious versatility of such hospital spaces, often unreferenced in psychiatric histories of these places.

## Carnegie Lodge and Adam’s workshop: carving, storing, sourcing

Carnegie Lodge, occupied by Clerk of Works Mr Orkney, became a significant site both for nurturing Adam’s artistic production and housing his stone heads. Orkney was particularly encouraging of Adam’s stonework, and Carnegie Lodge itself became a significant site for Adam, home to many of his stone creations. Other-worldly figures littered the Orkney family’s lawn, visitors being welcomed by a varied collection of Adam’s numerous stone heads.^5^ Angus sculptor William Lamb (1893–1951), arguably one of Scotland’s most important yet largely forgotten sculptors (Stansfield, 2013), was one such (frequent) visitor to the Lodge. Lamb was struck by Adam’s work and intrigued by his eccentric methods, and it is reported that Lamb offered him a set of his own chisels and hammers, an offer politely declined by Adam in favour of his own toolkit (Keddie, 1984: 71). As well as meeting in and around Carnegie Lodge, Lamb invited Adam to visit him at his own studio in Montrose. Glimpses of Adam carefully watching Lamb’s unusual technique were frequently noted, but he would duck out of site into the shadowy corners of the workshop whenever another visitor arrived.

The relationship between the two men demonstrates the importance of broadening asylum histories beyond the hospital wards to the houses and gardens within and beyond an asylum’s perimeter. Correlations can be made with research on sports activity in asylums, which has sought to place asylums at the centre of wider societal networks (Ellis, 2013). For Adam, significance was found in attachments to the immediate asylum landscape as source and site for creative activity, but spaces outwith the hospital, such as Lamb’s studio, also offered opportunities for learning his craft and being inspired. Recent research reveals the embeddedness of asylums within the social, political, moral and cultural geographies of the locales in which they are situated (Coleborne, 2010; Prior, 2012). By tracing Adam’s relationships with the individuals and spaces associated with Carnegie House, actors and sites effectively energized by the stone heads, a new species of asylum history is arguably written.

Adam’s increased stonework activities, coupled with his fear of being watched, led to him being offered his own workshop space at the hospital. Old storerooms housing remnants of a past asylum system now became a place for exploration, reverie and creativity. Wood (2000: 40), considering studios in art therapy, conjectures: ‘A studio is a place that can be returned to repeatedly for the ongoing process of making art … It is a place for working with the hands where mess can be tolerated at least some of the time … a suitable shelter for their approach to art making.’

The length of Adam’s incarceration highlights the transitional nature of the asylum experience and the destabilizing effects that external events can have for patients inside. Disruption to admissions, wages, staffing and ward structure, specifically due to war, altered the everyday experiences of the long-stay patients (THB 23/17/1 and 2). Rhodes (2000: 24) claims that the creation of outsider art can be ‘used as means of reasserting order on a chaotically changed world’; hence, as the world changed around Adam, he deployed his stone art as a stabilizing practice. The space demarcated as his workshop hence became a sanctuary over which he could exert some control, enforcing some measure of stasis. When discussing studio spaces, Hughes (1990, quoted in Wood, 2000: 41) remarks:

They are part of the solemn game of stasis. The essence of this place is that things do not change in it, except that dust accumulates, waste pigment slowly builds its reef on the floor, the light fluctuates … [I]t offers the painter a certain stability, a guarantee of changelessness … the refuge.

Alongside Adam’s gathering of discarded materials from the asylum grounds, Orkney also sourced materials for carving in the workshop. In 1928 the long-standing bridge over the River South Esk was being replaced and numerous unwanted stones were delivered to the hospital for building purposes (Keddie, 1984: 67). These stones became a rich source for Adam’s work, frequently making their way into the workshop to be transformed.

## Memories of stone: stone matters

Adam’s work demands attention not only because of its haunting nature, but also because of how the timeless nature of stone reflected his experience. For Adam, carving in stone can be directly related to his family and the embedded memories of his Shetland home. It may be argued that, by actively engaging in sculptural production and placement in referential places, Adam was exploring and memorializing elements of his lost familial past. Reflecting on his home in the early 1890s, before his incarceration to Montrose, Adam composed the poem ‘Shetland’ published in *The Shetland Times*:

North in the sea, remote from other lands,

Alone in silent majesty arise

The Shetland Isles: a place in history …

Home of my ancestors: thy moaning sea: (extract from Christie, 1891)

The hospital was frequently home to those experiencing mental health difficulties from the Shetland Isles (Presly, 1981), and Adam was the second of his immediate family to become a patient at Montrose. When Adam’s mother, Isabella, died on 20 June 1876, Adam was only nine years old and his household was thrown into life-changing grief. Lawrence, Adam’s father, became very disturbed and three months after the event was admitted to Montrose, far from his croft home in Aith (Keddie, 1984). Unlike Adam, Lawrence returned home apparently well after his treatment, but the fear that stalked a young Adam about his father’s disappearance to the asylum at Montrose must have held a lasting effect as he boarded the boat 22 years later, with two attendants, for the overnight journey to the mainland.

The confined space of the ship, travelling in darkness through the North Sea from Lerwick to Montrose, was the beginning of Adam’s asylum journey, but little is known about such experiences. Donoho (2012), reconstructing the historical geographies of insanity in the Scottish Highlands, reports that abuses on many of these journeys took on multiple physical and emotional forms. Examples given in the 1857 Lunacy Commission report tell of one patient travelling to Montrose who arrived ‘in so anaemic and exhausted a condition as to make recovery almost hopeless’ (in Donoho, 2012: 243).^6^ Many of the patients travelling to southern asylums were accompanied by the Sheriff’s officer or another non-medical expert, which could lead to misunderstanding of patients’ needs. Individuals might be restrained in some way, potentially for their own safety, making for a frightening and uncomfortable experience. Adam’s anxiety over his removal from his beloved homeland is not recorded, but the significance of such an experience is arguably etched into the stone faces left behind.

Similar to Adam’s own fate many years later and in a location far removed from the hospital, his mother Isabella was buried in an unmarked grave. The cemetery in Cunningsburgh was a disturbing place for Adam, and he grew increasingly angry about the lack of a lasting marker to his mother’s life and death. Discourses surrounding public memory recognize the significance for the remembering process of a physical place and symbolic space, with Wright (2005: 55) stressing that ‘[a] memory must have a place where a memory can crystallize and secrete itself’. The lack of visual markers can have devastating effects, and in his early twenties Adam decided to make amends for this lack of an eternal symbol. He walked 10 miles to the nearest quarry site to select a suitable stone and carried it back to his home, where he inscribed the stone to his mother and placed it on her final resting place (Keddie, 1984: 35). This act of deep love surely then resounded in Adam’s subsequent stonework, hinting at the emotionally-charged memories of home and family shaping, even driving, his obsessive carving of stone at the asylum.

In foregrounding such personal and specific asylum histories, it can be easy to romanticize the experiences of these patient-artists. As Maclagan (1987: 17) warns, it is crucial to heed the pain within which this creative impulse is intimately folded:

We can’t avoid the possibility that what drives Outsiders to create – what forces them as much as it leads them – is one form or another of underlying suffering. Sometimes this suffering is plainly deducible from their biographies. But it may be that a more obscure discomfort lies at the root of most creativity, that art itself manifests a certain uneasy nostalgia, a sense of not being quite at home in the world.

Keddie (1984: 58) states that on admission Adam was clearly suffering from psychosis and that previously he had been plagued by threatening voices. From his medical notes, it is plain that there were phases of his institutional life which were incredibly painful, with ideas of persecution and periods of restlessness leading him to be confined to his ground floor dormitory in the main hospital building (Keddie, 1984: 63). Montrose may have been known for its progressive treatment of the mentally ill, but it was still a place where incarceration was enforced and treatment was often distressing. Adam undoubtedly suffered sadness, pain and loneliness here, and his art must be seen as an expression of his life and creative talent, but also as a lasting reminder of the difficulties integral to his mental ill health and the challenging experiences frequently faced.

## Conclusions

For Dubuffet, the type of artwork discussed above aspires to be ‘an art which arises directly from this daily life, which would be a direct emanation from our real life and our real moods’ (Dubuffet, quoted in Heller, 1984: 22). By tracing the stories of stone through Adam’s head sculptures, a number of significant small sites and spaces of the Scottish asylum experience have been revealed. Casting the external spaces of asylum grounds as places of creativity, connection and hiding demonstrates the need for increased scholarship into the collective and individual creative expression of asylum small spaces. The journeys made by individuals within the asylum and the emotional memory spaces of home encapsulated by Adam’s sculptures all suggest the variety of real-world experiences to be mapped from Laing’s art collection. By opening up essential spaces for the production of Adam’s art, such as gardens and the hospital’s foliage corridors, it becomes evident that this work can offer a counterpoint to the surveillance culture of asylumdom. The personal and more historically symbolic function of Adam’s stone heads is shown here to offer a material, emotional and creative counter-culture that subverts certain key tenets of surveillance culture in such spaces. Inquiring into such patient artwork paves the way for new species of asylum histories to be written.

Using material from the Art Extraordinary collection to disclose spaces of asylum experience leads to questions about what role such art can play in how we know and understand our worlds. Focusing on the artwork itself, following the stories of stone, allows a new set of patient experiences to be illuminated that is currently missing in the stories of the insane, as well as highlighting the liminal place that outsider art, including Art Extraordinary, has occupied in such scholarship. The spatial and material dimensions of recovery have also been explored, detailing the experiential reworking and memorialization of experience by patients – in Adam’s case through stone, but also hinting to those other individuals whose work, perhaps, remains unknown. By unravelling the histories associated with the collecting of Art Extraordinary, the importance emerges of individuals such as Joyce Laing and their changing conceptions of value, signalling complex entanglements among objects, collectors, social relations and time in the asylum context. Maclagan (1987: 8) suggests that the ‘secrecy’ of outsiders is related to where they work, an ‘in-between territory’ that holds within its sphere a silent indication of something that cannot be shared. However, this paper has begun to show how historians (and historical geographers) can usefully chart this no-person’s land using the artwork as a focus, illuminating the contextually sited experience of individual patient-artists and their worlds. It has sought to open up new pathways for addressing outsider art collections in histories of psychiatry, identifying a specific Scottish realm of interest for future study.

## Notes

1. A contentious issue is the place of the artist-patient in the representation of the work.

2. ‘Outsider Art’ was first coined by Roger Cardinal in his 1972 book entitled *Outsider Art*.

3. Throughout I will refer to Adam Christie as Adam. This is in no way to devalue his artistic contribution, but instead, arguably, allows the reader to remain closer to his personal experiences of being-in-place.

4. This passage of creative writing is from the author’s research diary during a visit to the hospital site in Montrose in 2015.

5. It is difficult to be accurate about the total number of stone heads created by Adam, as many were destroyed and some remain hidden. However, it is estimated that over 200 sculptures were created.

6. Donoho (2012) also gives examples where patients were treated well on their journey to the asylum.
